# Alveolar cells in the mammary gland: lineage commitment and cell death

**DOI:** 10.1042/BCJ20210734

**Published:** 2022-05-12

**Authors:** Christine J. Watson

**Affiliations:** Department of Pathology, University of Cambridge, Tennis Court Road, Cambridge CB2 1QP, U.K.

**Keywords:** binucleate, cathepsins, cell death, lineage, mammary gland, STAT3

## Abstract

The mammary gland provides a spectacular example of physiological cell death whereby the cells that produce milk during lactation are removed swiftly, efficiently, and without inducing inflammation upon the cessation of lactation. The milk-producing cells arise primarily during pregnancy and comprise the alveolar lineage that is specified by signalling pathways and factors that are activated in response to pregnancy hormones. There are at least two alveolar sub-lineages, one of which is marked by the presence of binucleate cells that are especially susceptible to programmed cell death during involution. This process of post-lactational regression, or involution, is carefully orchestrated and occurs in two phases, the first results in a rapid switch in cell fate with the secretory epithelial cells becoming phagocytes whereupon they destroy dead and dying cells from milk. This reversible phase is followed by the second phase that is marked by an influx of immune cells and a remodelling of the gland to replace the alveolar cells with re-differentiated adipocytes, resulting in a return to the pre-pregnant state in preparation for any subsequent pregnancies. The mouse mammary gland provides an excellent experimental tool with which to investigate lineage commitment and the mechanisms of programmed cell death that occur in a normal physiological process. Importantly, involution has highlighted a role for lysoptosis, a mechanism of cell death that is mediated by lysosomal cathepsins and their endogenous inhibitors, serpins. In this review, I discuss alveolar lineage commitment during pregnancy and the programmed cell death pathways that destroy these cells during involution.

The mammary gland at birth is a rudimentary structure with a small arbourised ductal tree embedded within a fatty stroma [1]. Growth is commensurate with body growth until puberty when club-like structures called terminal end buds (TEB) arise at the tips of ducts. TEB are the site of stem and progenitor cell expansion and ducts elongate until the limits of the fat pad are reached whereupon the TEB regress. The ductal epithelium is bilayered comprising an outer layer of contractile basal/myoepithelial cells and an inner layer of luminal cells [1]. Basal cells can be marked by their expression of the intermediate filament keratin 14 (K14) and the transcription factor p63. The luminal cells may be either estrogen hormone receptor alpha-expressing/progesterone hormone receptor-expressing (ER^+^/PR^+^) or non-expressing and are marked by the expression of keratin 8 (K8) which may be present at high or low levels [2].

The cyclical development of the mammary gland, with the massive expansion of cells that occurs during each and every pregnancy, requires the presence of stem cells and/or long-lived progenitors to produce the significant numbers of progeny that are required to differentiate and produce milk. There has been much interest in mammary stem cell (MaSC) research over the past decade or so and we now have a very good understanding of the different MaSCs and progenitors that are required to generate a functional mammary gland [1,3]. Foetal (f) MaSCs arise in the mid-gestation embryo and although initially bipotent, only rare and primarily quiescent bipotent cells remain after birth, with the remainder becoming progressively lineage restricted. Thus, most adult MaSCs are lineage-restricted under normal homeostatic conditions although they can be reprogrammed to a bipotent or multipotent state if transplanted into an intact mammary gland or fat pad that has been cleared of endogenous epithelium [4–6].

In the following sections, I will provide an overview of lineage commitment during pregnancy, the generation and role of binucleate cells during lactation, and the events that occur during involution, with a focus on programmed cell death.

## Lineages arising during pregnancy

During pregnancy, the expansion of the rudimentary ductal tree that is present at birth, involves extensive tertiary branching and the development of lobuloalveolar structures at the tips of these branches. These lobuloalveoli are comprised of a similar bi-layered structure to the ducts with an outer myoepithelial layer and an inner luminal layer, the luminal alveolar cells producing milk during lactation with the basal/myoepithelial cells producing the contractile force to expel milk into the hollow lumen of the alveolus.

The levels of the steroid hormone progesterone (P), and the polypeptide hormone prolactin (Prl), rise during pregnancy and this triggers tertiary branching and alveologenesis and is associated with extensive proliferation, followed by differentiation. A number of gene deletion studies have demonstrated that Signal transducer and activator of transcription 5 (Stat5) [7], Elf5 [8], Stat6 [9] and RANKL [10] are required for this proliferative growth while PR is necessary for ductal side-branching and alveologenesis [11,12]. The two types of luminal cells that constitute growing lobuloalveolar structures are comprised of cells that are either hormone sensing ER^+^/PR^+^ (and often co-express Gata3), or ER^−^/PR^−^ hormone responsive (and often co-express pStat5/Elf5) [13]. Notably, the zinc finger transcriptional repressor Blimp1 has been shown to be expressed in progenitor cells that give rise to the proliferative pStat5/Elf5^+^ cells [14,15] and the Krüppel-associated box (KRAB) zinc finger protein Zfp157 (also known as Roma) controls the balance of these different lineages [13]. Thus, there are at least two distinct lineages of alveolar cells that arise during pregnancy and that co-operate to generate lobuloalveolar structures.

As pregnancy progresses, alveolar cells express different subsets of genes, many of which are regulated in a chronological order by the Prl-regulated transcription factor Stat5 [16]. Unsurprisingly, the expression of milk protein genes is massively induced with β-casein (Csn2) and whey acidic protein (Wap) being up-regulated more than 1000-fold as a consequence of the presence of mammary-specific super-enhancers [17]. Furthermore, promoters of genes that bind Stat5 have histone H3 lysine 4 trimethylation (H3K4me3) marks, an epigenetic mark of active transcription, when lactation commences. It is worth noting that lactation is held in check by high levels of progesterone and can commence before the normal completion of pregnancy should progesterone levels fall precipitously.

Considerable insights into mammary gland development and lineage specification have been gained with the development of sophisticated mouse models for lineage tracing coupled with a variety of new approaches including single cell RNA sequencing (scRNA-Seq), epigenetic analyses, imaging in 3D and in live animals, and using lentivirus to barcode embryonic mammary precursors. These new experimental approaches have revealed that ∼120 bipotent fMaSCs are sufficient to generate an adult mammary gland [18] and that even a single fMaSC can contribute extensively to postnatal development [19]. Of particular utility is lineage tracing using specific promoters to drive Cre-mediated recombination in reporter mice including the multicolour Confetti mouse [20–22] and the *R26^[CA]30EYFP^* ‘slippage’ mouse model [2].

## Lineage tracing of alveolar progenitors

Lineage tracing, using genetic markers, has been utilised to investigate the contribution of stem and progenitor cells to lineages at all stages of mammary gland development including embryonic, perinatal and pubertal (reviewed in [3,23,24]) and during pregnancy and lactation [25]. The firm conclusion is that both luminal and basal lineage progenitors are unipotent as are the majority of MaSCs. Blimp1 is induced in a subset of alveolar cells during pregnancy and, as mentioned above, these cells proliferate to generate pStat5/Elf5^+^ cells (that are ER/PR negative) and are maintained as long-lived alveolar progenitors. There is evidence that progenitor cells that give rise to the alveolar lineage are already specified in the post-natal gland but do not expand until pregnancy [26]. Another such population, so-called parity-identified mammary epithelial cells (PI-MECs), are lobule-restricted progenitors that contribute only to the ER^−^ luminal alveolar population and were identified using the whey acidic protein (WAP) promoter to drive Cre expression in Rosa26-lox-Stop-lox-YFP mice [27]. Notch 2 identifies a lineage that is required for alveologenesis with notably only one or two marked luminal cells per alveolus [28].

The *R26^[CA]30EYFP^* ‘slippage’ mouse model relies on a DNA replication error to put a reporter gene in frame and is a rare event, resulting in a single labelled clone per mammary gland. Analysis of lobuloalveolar structures in lactating mice with the *R26^[CA]30EYFP^* mouse revealed a variety of clonal patterns. While some alveoli were comprised almost completely of labelled cells, others had only some labelled progeny suggesting that at least two progenitors had contributed daughter cells to the alveolar structure [2]. Consistent with other studies, clonal progeny were restricted to either the luminal or basal lineage. Interestingly, one clone was observed that had a labelling pattern reminiscent of that seen with Notch 2 lineage tracing where only one, or occasionally two, luminal cells were marked per alveolus [2,28]. The role of such a cell, that does not seem to proliferate, can only be speculated upon. However, luminal alveolar progenitors may reside in a niche that maintains their viability during a subsequent involution. Since ablation of Notch 2-expressing lineages in the pubertal gland impaired the formation of alveolar clusters, and a subset of Notch 2-expressing cells co-localise with the origin of tertiary branching [28], it can be speculated that a Notch 2-expressing cell may have a role to play in maintaining this niche.

While the myoepithelial/basal cells in ducts are elongated and aligned in parallel, those in alveoli form a basket-like network over the surface of the luminal alveolar layer, that encircles a hollow lumen [29]. It is an open question whether the extensive proliferation of the basal cells is required or whether basal cells at the branch tips stretch out over the expanding alveolus. However, lineage tracing reveals that at least two progenitors are probably required. Thus, alveoli are formed by the cooperative growth of at least three lineages: basal, ER^+^/PR^+^, and ER^−^/PR^−^, the last category providing the majority of cells. Note that while luminal cells are either ER^+^/PR^+^ or ER^−^/PR^−^, these two steroid hormones play quite different roles in the development of the mammary epithelium during pregnancy. PR is an ER target gene [30] and P is essential for proliferation, ductal side-branching and alveologenesis [31]. Moreover, P controls proliferation of ER^−^/PR^−^ cells in a paracrine fashion through receptor activator of nuclear factor κB ligand (RANKL) [32]. The *PR* gene has two promoters with the shorter PR-A isoform being required for alveologenesis, which occurs independently of RANKL and Wnt4, while the PR-B isoform is required for ductal side branching [33]*.* Lineage tracing studies have clearly shown that the ER^+^ and ER^−^ lineages are maintained by distinct lineage-restricted progenitors [34].

The advent of scRNA-Seq studies of mammary cell populations has revealed further lineage complexities [35] and there is a plethora of studies using scRNA-Seq to characterise and identify different subpopulations of mammary epithelial cells at different stages of development [36–38]. The authors of these studies do not come to the same conclusions, probably reflecting the different sequencing platforms and analytical tools utilised. Using a pseudotime trajectory analysis, Bach and colleagues identified 15 clusters and posit the existence of a luminal progenitor that can give rise to intermediate, restricted alveolar and hormone-sensing progenitors that subsequently undergo changes in their transcriptome in response to the pregnancy cycle [36]. A similar finding has been reported also using scRNA-seq [38] and chromatin using scATAC-seq [39]. In contrast, Pal and colleagues suggest that gene expression in the pre-pubertal epithelium shifts from a basal-like programme to distinct lineage-restricted programs in puberty [37]. Importantly, scRNA-seq studies have identified a surprising number of cellular intermediates, particularly in the luminal compartment, which may be short-lived transit amplifying lineage-committed cells that are not detected by genetic lineage tracing studies.

## Lactation and polyploidy

During late gestation, the gland is poised to make milk as soon as required, although high levels of P inhibit milk secretion. This is particularly important for humans where premature birth may occur and ensures that breast milk can be produced. The precipitous drop in P at birth triggers a final round of proliferation and the fat pad is filled with alveolar epithelium, replacing differentiated adipocytes.

Milk provides all the energy and nutrients required for the newborn to thrive and so lactation imposes a high demand on the alveolar cells to produce protein and fat. The expression of milk protein genes is dramatically increased and in order to achieve the high levels of protein production required, the translational machinery of the cell needs to be enhanced. One mechanism would be to increase the number of ribosomal genes. Interestingly, historical studies from four decades ago suggested that full differentiation of secretory alveolar cells ‘requires DNA synthesis inconsequent of mitosis’ and that this could involve polyploidy [40]. This work was not followed up until recently when it was reported that binucleate cells could be observed in lactating mammary glands of mice, marsupials, cows and humans [41,42], the presence of binucleate cells suggesting a failure of cytokinesis. Polyploidy is seen in trophoblast giant (TG) cells and megakaryocytes, which have DNA contents between 8N to 64N and is a consequence of endoreduplication in TG cells and endomitosis in megakaryocytes [43].

Notably, the number of binucleate cells can be increased by deleting Zfp157/Roma, which is a transcriptional target of Stat6 [13]. When Zfp157 is absent, alveolar cells undergo enhanced proliferation that skews the ratio of luminal alveolar pStat5 cells to Gata3 cells, with the number of the latter being diminished [13]. At day 10 lactation, when cells are normally terminally differentiated, Zfp157 deficient luminal cells continue to undergo DNA replication, without cell division, increasing the number of binucleate cells that exhibit evidence of substantial DNA damage [42]. Immunofluorescence analysis of 10 day lactation Zfp157 deficient glands revealed the presence of γH2AX foci and large 53BP1 foci that suggest replication stress-mediated DNA lesions have arisen in the previous S phase [42,44]. It is intriguing that these ‘damaged’ cells continue to make milk and survive. Normally, when Gata3 is deleted, lactation fails but this can be overcome by co-incident deletion of Zfp157 although the mechanism by which knockout of Zfp157 compensates for Gata3 deficiency is not clear [13]. However, in this context it is interesting to note that many transposable elements (TE) contain binding sites for ERα and Gata3 [45] and that KRAB domain zinc finger proteins are involved in suppressing the activity of TE [46].

A number of kinases are associated with the mitotic spindle and mitosis, one of which is Aurora kinase A. When this kinase is conditionally deleted in mammary gland, basal/myoepithelial cells are unaffected, while there is a dramatic reduction in the number of binucleate luminal cells. This has severe consequences for the suckling pups which fail to thrive 4 days after birth when suckled by Aurora A deficient dams [41]. This genetically confirms that binucleate cells are essential for full functional differentiation of the mouse mammary gland and milk production. It is interesting that not all cells are binucleate and this raises the possibility that a balance must be achieved between viable, undamaged cells and those that are binucleate and thus destined to die at the end of lactation due to their harbouring damaged DNA [42].

Lactation is dependent on the survival of secretory alveolar cells. The anti-apoptotic protein myeloid cell leukemia 1 (Mcl-1) (see cell death section below) is strikingly up-regulated at the lactation switch and is required to maintain the survival of cells during lactation [47] while the related protein Bcl-x_L_ is not [48]. The membrane receptor Butyrophilin 1A1 (BTN1A1), that is involved in the secretion of lipid droplets from secretory mammary epithelial cells, is required also for their survival with a considerable proportion of cells dying in *Btn1a1* knockout mice [49]. Furthermore, ablation of ephrinB2, a ligand for the receptor tyrosine kinase EphB4, results in lactation failure and precocious involution [50].

## Involution and cell death

As mentioned above, when milk is no longer required and suckling ceases, the lobuloalveolar structures regress and the mammary gland is remodelled to resemble the arbourised ductal tree that was present before pregnancy. The entire cycle of alveologenesis, lactation and involution takes place with each and every pregnancy and thus must be exquisitely controlled to maintain functionality. The involution process is triggered by milk accumulation and characterised by two phases of extensive programmed cell death, an initial reversible phase that lasts for ∼48 h in the mouse and results in the shedding of dead luminal cells into the lumen of the alveolus, which notably does not collapse, and a second phase that results in the death of the surviving alveolar cells coupled with alveolar collapse, remodelling of the extracellular matrix, influx of immune cells and redifferentiation of the adipocytes in the stroma [51,52]. Involution is usually a gradual process in both animals and humans but a synchronous involution can be initiated experimentally by removal of the suckling pups (usually at day 10 of lactation in the mouse, the peak of lactation) or by sealing the teats with veterinary glue [51]. The latter approach is particularly useful for investigating locally acting, and not systemic, factors [53].

## Mechanisms of programmed cell death

Cell death is important during embryonic development for tissue sculpting and organogenesis and is essential in maintaining cellular homeostasis in adult organisms [54]. Importantly, cell death must be tightly controlled as excessive cell death can lead to degenerative diseases while insufficient death of damaged cells can result in cancer. In the mammary gland, initial studies on the mechanism of cell death during involution focussed on apoptosis [55] as this was the primary mechanism investigated in the early 1990s. Apoptosis, first described 50 years ago by Kerr, Wyllie and Curry [56] is characterised by activation of a set of specific cysteinyl aspartate proteases called caspases, that cleave target intracellular proteins resulting in distinctive morphological features and causing the demise of the cell. There are two distinct apoptotic pathways initiated by different upstream signals and caspases [57]. The extrinsic pathway is induced by caspase 8, downstream of death receptors (DRs), while the intrinsic pathway is induced by caspase 9 activation that is initiated by mitochondrial outer membrane permeabilization (MOMP) and cytochrome c released to the cytosol, downstream of B-cell lymphoma 2 (Bcl2) family proteins [47,58]. This family of proteins can have pro-survival (Bcl-2, Bcl-x_L_, Bcl-w, Mcl-1 and A1/Bfl-1) or pro-apoptotic (Bax, Bak, Bok and multiple BH3-only domain proteins including Bim, Bid and NOXA) activity and their balance determines whether MOMP takes place. Regardless of the initiating pathway, apoptosis is completed by the three executioner, or effector, caspases 3, 6 and 7. It is worth noting that caspase 3 and caspase 7 can usually compensate for the absence of each other but caspase 6 seems to have some distinct functions. Apoptosis is generally a non-immunogenic process as the cell ‘corpses’ are removed by professional phagoctyes and destroyed.

In the past decade, there has been an explosion of interest in alternative pathways of programmed cell death and at least 12 genetically programmed pathways have been identified that induce cell death in response to different signals and different situations [59]. Many of these pathways, including intrinsic apoptosis, extrinsic apoptosis, necroptosis, ferroptosis, pyroptosis, parthanatos, entotic cell death, NETotic cell death, lysosome-dependent cell death, autophagy dependent cell death, mitochondrial permeability transition-driven necrosis, immunogenic cell death, cellular senescence, and mitotic catastrophe can be defined in molecular terms [59]. The most widely studied, and most frequently observed, pathways in addition to apoptosis are necroptosis and pyroptosis, both forms of regulated necrosis [60]. During necroptosis, receptor interacting protein kinase 1 (RIPK1) activates RIPK3 which in turn phosphorylates its substrate, mixed lineage kinase domain like pseudokinase, that inserts in the plasma membrane causing damage and leakage of cellular constituents. Pyroptosis is another caspase regulated pathway induced by the inflammatory caspases 1 and 11 in mouse, and caspases 4 and 5 in humans. These caspases cleave Gasdermin D (GSDMD) to produce an N-terminal fragment that forms a plasma membrane pore, with consequent leakage of cellular contents while caspase 1 also cleaves the precursor cytokines pro-IL-1β and pro-IL-18 to produce inflammatory mediators [57].

Recent evidence indicates that receptor interacting RIPK1 and caspase-8 are the primary regulators of the interaction between these three important cell death pathways and these interactions dictate the outcome of cell death signalling and the type of cell death induced [61].

## First phase involution and cell death

**Figure 1.**
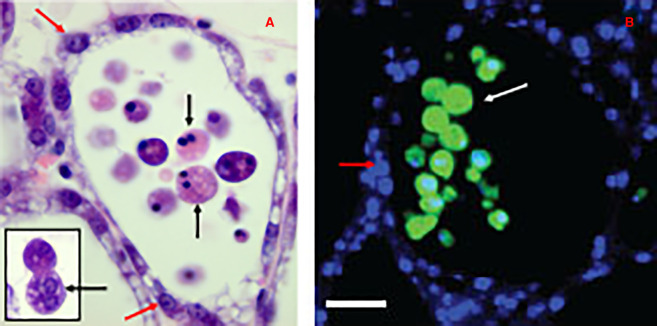
Morphology of detached and dying cells at 24 h involution. (**A**) shows an H&E stained thin section of tissue from a 24 h involution mouse mammary gland; a single alveolus is shown. Black arrows point to cells that have detached from the alveolar epithelial wall but are still intact and exhibit two hypercondensed nuclei. Red arrows point to viable luminal cells that are still within the alveolar epithelial wall. The basal/myoepithelial cells are too thin to be detected. The insert shows the lack of nuclear condensation in glands deficient for caspase 3. (**B**) shows a single alveolus immunostained for cleaved (active) caspase 3 in green (white arrows) and nuclei with Hoechst in blue (red arrows). Note the large number of shed cells staining for cleaved caspase 3 in the lumen but none in the alveolar wall.

Given the many possible mechanisms of cell death induced early in the involution process, clues were sought as to possible pathways. During involution, it was observed that cells are shed into the alveolar lumen, beginning at 12 h following forced involution, and that these cells remain intact. Furthermore, only the shed cells stain positively with antibodies for cleaved caspase 3 with cells in the alveolar wall not exhibiting caspase 3 activity until 72 h involution (Figure 1). This suggests that the initial wave of cell death may not be apoptosis and that caspase 3 cleavage could be induced in detached cells by anoikis. Ablation of the executioner caspases 3, 6 and 7 further suggested that apoptosis is not required for first phase cell death [62] since involution progressed normally in the absence of executioner caspase activity. However, in the absence of caspase 3, the nuclei in shed cells do not condense possibly due to the absence of caspase 3-mediated cleavage of Inhibitor of Caspase Activated DNAse (ICAD). It is notable that most shed cells are binucleate suggesting that these cells may be more susceptible to the induction of cell death than mononucleate cells. This is not surprising since there is considerable elevation of DNA damage markers in binucleate cells [42]. Taken together, these observations suggested that apoptosis is not the initial mechanism of cell death during involution.

Insights into the programmed cell death pathway(s) utilised during involution were gained from observations on the dramatic activation of Stat3, another member of the Stat family of transcription factors, within 12 h of forced involution [63]. This prompted studies on the conditional deletion of Stat3 during lactation using a milk protein gene promoter to drive expression of Cre recombinase in the alveolar epithelium [64]. Remarkably, cell death was substantially diminished in the absence of Stat3 and the gland failed to regress for the first 3 days [65]. Using these knockout mice to analyse Stat3 target genes at 24 h involution revealed a number of Stat3 target genes including the lysosomal hydrolases cathepsin B and cathepsin L that are up-regulated by Stat3 while expression of the endogenous cathepsin inhibitor Serpina3G (known also as Spi2a) is dramatically reduced 60-fold in the presence of Stat3 activity [62]. This suspected role of lysosomal enzymes in the cell death process during involution led to an investigation of the ‘leakiness’ of lysosomes that had been isolated from mammary glands at day 10 lactation and 24 h involution. While lysosomes isolated from lactating glands remained intact, those isolated from involuting glands leaked cathepsin B and cathepsin L [62]. These results led to the conclusion that during first phase involution, luminal alveolar cells die via a lysosomal-mediated pathway of programmed cell death (LM-PCD). Subsequent work demonstrated that milk fat globules (MFG) are taken up from the lumen by alveolar cells, a process that is enhanced by pStat3, where the MFG lipids are digested in lysosomes to their constituent fatty acids. The consequent elevation of the levels of oleic acid could possibly perturb the lysosomal membrane causing its permeabilization and thereby facilitating the release of cathepsins that kill the cell by digesting cellular constituents in a manner similar to executioner caspases [62,66]. Stat3 also modulates trafficking of proteins such as annexins and flotillins from the plasma membrane to the lysosome [67] where they may affect its function. It is worth noting that historical electron microscopy studies had suggested changes in the lysosomal compartment during involution with cathepsin D being released from lysosomes [68–70]. More recently, cathepsin D has been shown to be processed and active during involution [71,72].

CRISPR–Cas9 mediated deletion of the gene for Stat3 in EpH4 mouse mammary epithelial cells abolished the up-regulation of cathepsin B expression in response to oncostatin M stimulation, a potent induced of Stat3 activity [67]. Interestingly, the zinc transporter ZnT2 likely also plays a role in lysosome biogenesis and lysosome-mediated cell death during involution [73], as confirmed using ZnT2 knockout mice. These mice had impaired alveolar regression that was associated with reduced levels of pStat3. Furthermore, assembly of the vacuolar ATPase, that is essential for maintaining the intracellular pH of lysosomes, was inhibited in ZnT2 knockout mice and this resulted in smaller, and fewer, lysosomes.

Since milk contains significant amounts of calcium (Ca^2+^), involution will cause transport of Ca^2+^ into milk to cease and levels in the alveolar cell to rise. This increase in cytosolic Ca^2+^ levels is likely to activate calpains, Ca^2+^-sensitive non-lysosomal cysteine proteases [74]. During lactation, the calcium pump ATPase 2 (PMCA2), which is located at the apical surface of the plasma membrane, transports calcium into milk. PMCA2 interacts with NHERF1, the PDZ domain-containing scaffolding molecule sodium-hydrogen exchanger regulatory factor, levels of which are up-regulated during lactation [75]. At the onset of involution, expression of both PMCA2 and NHERF1 are down-regulated and leads to lysosome-mediated cell death.

An important signalling pathway that regulates the survival/death decisions is the nuclear factor (NF)-κB/IκB kinase (IKK)/DR pathway [76] that is upstream of both apoptosis and necroptosis. NF-κB was shown to be inactive during lactation but dramatically up-regulated at the onset of involution where is exerted a survival function [77]. Conditional deletion of IKKβ/2 resulted in a significant delay in involution possibly through transcriptional regulation of the DR ligands tumour necrosis factor (TNF)α and tumour necrosis factor-like weak inducer of apoptosis (TWEAK) [78].

It is clear that pStat3 and a variety of other factors are important regulators of elevated lysosome biogenesis at the onset of involution [73]. This is an interesting counterpoint to lysosome biogenesis in a starvation situation where the transcription factor EB (TFEB) has the primary role [79]. During involution, the alveolar cells are not subject to starvation but to nutrient excess arising from the uptake of MFG and other milk constituents.

Recent work in mouse and human epithelial cell lines has shown that intracellular serpins, particularly Serpinb3a/SERPINB3, can protect from cell death mediated by lysosomal membrane permeabilisation and cathepsin activity. The authors suggest that this cathepsin L/serpin regulated pathway be called lysoptosis [80]. Given that LM-PCD during mammary gland involution requires cathepsin L and down-regulation of Serpina3G, I suggest that the term lysoptosis be adopted for cell death during early mammary gland involution rather than LM-PCD, the terminology that we used for our work on lysosomal cell death [62,66].

The first 48 h of involution is a dynamically regulated process whereby the lysosomal compartment is enhanced and the fate of the alveolar luminal epithelial cells is switched from milk secretion to phagocytosis, resulting in the delivery of MFGs to lysosomes. It is more difficult to study the fate of the myoepithelial cells as they are so thinly stretched during involution and although it has been assumed that they die along with the luminal cells, recent deep imaging studies suggest that they may not die but simply contract and shrink back towards the ductal tree [29] and do not die. If this supposition is correct, then the network of myoepithelial cells that surrounds each alveolus may arise from ductal myoepithelial cells that adopt a different shape when in contact with alveolar epithelium.

## Second phase involution, cell death and destruction

The switch to second phase involution occurs ∼42 h after forced involution in the mouse (Katherine Hughes, PhD thesis University of Cambridge). After this time point, the alveoli start to collapse and the ‘space’ in the gland is filled by re-differentiating white adipocytes. Indeed, measuring the area occupied by adipocytes is a useful measure of the extent of involution [81]. Recent studies have used the adiponectin promoter to genetically trace mature adipocytes and the results suggest that hypertrophy is the primary mechanism of adipocyte regeneration with milk-derived lipids being trafficked to adipocytes [82]. Concomitantly with adipocyte regeneration, the extracellular matrix is remodelled by matrix metalloproteases (MMPs) and serine proteases, activating plasminogen and causing detachment of alveolar cells, resulting in a second wave of cell death [52].

Gene expression studies at a number of involution time points revealed subsets of genes that are induced at distinct times during involution [83,84]. Four distinct transcriptional profiles are present in the first 4 days of involution, whereas there are 3 distinct profiles in lactation. At the peak of lactation (day 10 in mouse), in excess of 400 genes reach their peak expression before dramatically declining by 12 h of involution. A reciprocal pattern was observed for ∼500 genes that were specifically up-regulated within the first 12 h of involution. A further three sets of genes showed delayed up-regulation peaking at 24 h, 48 h, or 4 days, many of this last category showing a more gradual rise in expression. This indicates a series of sequential changes throughout the first and second phases of involution. Furthermore, there are a number of genes that are sharply up-regulated at the switch to the irreversible phase around day 2 including chitinase 3-like 1 (Chi3L1), a gene associated with wound healing [85].

The second phase of involution requires the participation of professional phagocytes to remove residual milk and cellular debris and there is a marked influx of immune cells at 72 h involution [29,86–88] that add to the population of resident ductal macrophages that are required to clear dead alveolar cells at this stage of involution [89]. Notably, the secreted glycoprotein milk fat globule epidermal growth factor (EGF) factor 8 (MFG-E8), which binds to cells that have flipped phosphatidylserine to the outer membrane leaflet, is required for second phase involution and the clearance of MFG [52]. Dead and dying cells accumulate in involuting mammary glands deleted for the receptor tyrosine kinase MerTK [90] suggesting that MerTK-mediated efferocytosis acts to prevent inflammation that would arise from the rupture of accumulated dead cells.

The mode of cell death during second phase involution is difficult to determine as most mouse models abolish first phase involution. Given the patterns of gene expression in second phase, and the continued activation of Stat3, it is possible that necroptosis is involved. Interestingly, however, caspase 7 is specifically cleaved at 72 hours involution suggesting that apoptosis may be involved [62]. It was noted in mammary glands conditionally deleted for Stat3 that p53 and p21 are up-regulated [91]. This prompted the double ablation of Stat3 and p53 which revealed a remarkable further delay in involution, with glands at day 17 involution exhibiting retained milk and some alveolar structures. This suggests that p53 mediates a default programme of cell death in the absence of Stat3 [92]. These observations, coupled with TUNEL positivity at 72 h involution, could suggest that apoptosis has a role to play in second phase involution. However, it is possible that necroptosis or another mechanism of cell death could be involved.

It is worth noting that both Stat3 and NF-κB, in addition to regulating cell death, regulate the acute phase response and inflammatory signalling [93]. It is remarkable that the extensive cell death and tissue remodelling that takes place in second phase cell death does not cause inflammation.

The mechanisms that ensure survival of alveolar progenitors to enable further pregnancy/lactation/involution cycles is not known. Ducts remain and it is possible that there is a reservoir of alveolar progenitors residing in a protective niche at branch points, possibly controlled by β-catenin responsive cells [94]. Notch1-expressing progenitors survive multiple rounds of involution where they contribute only to the ER^−^ lineage [26] as do PI-MECs [27]. The ER^+^ progenitor lineage is distinct and survives involution to regenerate only the ER^+^ alveolar lineage [34]. One striking difference between ductal and alveolar luminal cells is the expression of K8, with very few alveolar cells expressing detectable levels of K8 [2]. Interestingly, K8 has been shown to provide some resistance to DR-mediated cell death and so high levels of K8 expression may allow progenitors to survive [95]. Furthermore, alveolar cells switch fate upon the initiation of involution and take up MFG, thus ensuring their own demise by lysoptosis, while progenitor cells presumably do not as they are undifferentiated.

## Conclusion

**Figure 2.**
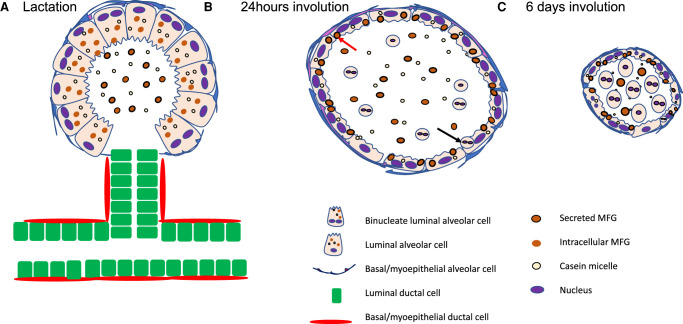
Schematic representation of the structure and morphological changes in mammary gland during lactation and involution. (**A**) The lactation diagram shows a duct and tertiary branch with a single lobuloalveolar structure. The luminal ductal cells are derived primarily from long-lived progenitors that may be either hormone receptor positive (ER^+^/PR^+^) or hormone receptor negative (ER^−^/PR^−^), and the basal ductal cells are derived from lineage restricted unipotent progenitors. Alveolar luminal cells derive primarily from ER^−^/PR^−^ progenitors while basal cells may derive from the ductal layer or be generated *de novo* from alveolar-specific progenitors. Myoepithelial cells are thinly stretched around the clusters of luminal cells and have a stellate structure. There may be a niche at the tips of tertiary branches that protects progenitors from cell death during involution (see text). The presence of binucleate cells, that have undergone a second round of DNA replication but have not divided, is indicated. Large milk fat globules (MFGs) are secreted, enveloped by the plasma membrane, while the milk protein caseins are secreted as micelles. (**B**) This diagram depicts a single alveolus at 24 h following forced involution. Activation of Stat3 initiates a fate switch in the secretory alveolar cells that now take back up secreted MFGs (indicated by red arrow) and shed cells (indicated by black arrow). This first phase of involution is reversible but after around 48 h, involution cannot be halted and the gland regresses almost completely to a pre-pregnant state. (**C**) By 6 days of involution, the alveoli have dramatically collapsed due to the extensive death of the luminal cells and remaining alveoli shrink back towards the ducts.

We now have a good understanding of the genetic mechanisms of alveolar mammary epithelial cell lineage specification during pregnancy and its subsequent destruction by programmed cell death during post-lactational regression (Figure 2). While many pieces of the involution jigsaw are now in place, there are a number of outstanding questions that will need to be addressed in future work. Why do some cells become binucleate during lactation and are these a different lineage? How is the balance of lineages determined? What are the earliest signals that initiate cell death? Why do only a proportion of alveolar luminal cells die in the first wave? What is the mechanism of transition from reversible to irreversible phase and is this related to the influx of immune cells? Which pathways of cell death in addition to lysoptosis participate in the involution process? How are the stem/progenitor cells protected from cell death during involution? These are challenging questions but the tools to answer them are now available and the next decade should be an exciting time for cell death research in the mammary gland.

## Data Availability

There are no original data in this manuscript.

## Abbreviations

BTN1A1: Butyrophilin 1A1
DR: death receptor
KRAB: Krüppel-associated box
LM-PCD: lysosomal-mediated programmed cell death
MFG: milk fat globules
MOMP: mitochondrial outer membrane permeabilization
RANKL: receptor activator of nuclear factor κB ligand
RIPK1: receptor interacting protein kinase 1
TE: transposable elements
TEB: terminal end buds
TG: trophoblast giant
Wap: whey acidic protein
